# Correlation between benign joint hypermobility syndrome and headache in children and adolescents

**DOI:** 10.1186/s12891-024-07473-3

**Published:** 2024-05-02

**Authors:** Mohsen Jari, Sogol Alesaeidi

**Affiliations:** 1 Department of Pediatric Rheumatology, Imam Hossein Children’s Hospital, Isfahan University of Medical Sciences, Isfahan, Iran; 2 School of Medicine, Isfahan University of Medical Sciences, Isfahan, Iran

**Keywords:** Benign joint hypermobility syndrome, Headache, Children

## Abstract

**Background:**

Benign Joint Hypermobility Syndrome (BJHS) is a most common hereditary connective tissue disorders in children and adolescents. This study aimed to investigate the prevalence and subtypes of headache in children with BJHS.

**Methods:**

This observational-analytical study was conducted in a case-control setting on school children aged 7 to 16 years in 2021–2023 in Isfahan, Iran. Students were examined for BJHS using Beighton criteria by a pediatric rheumatologist. Headache disorder was diagnosed according to the Child Headache-Attributed Restriction, Disability, and Social Handicap and Impaired Participation (HARDSHIP) questionnaires for child and adolescent and International Classification of Headache Disorders (ICHD-III).

**Results:**

A total of 4,832 student (mean age 10.3 ± 3.1 years), 798 patients with BJHS and 912 healthy children were evaluated. The probability of headache in children aged 7–11 with hypermobility was 3.7 times lower than in children aged 12–16 with hypermobility (*P* = 0.001). The occurrence of headache in children with BJHS was more than the control group (*P* = 0.001), and the probability of headache in children with BJHS was 3.7 times higher than in healthy children (*P* = 0.001). Migraine was the most common headache type reported of total cases. The probability of migraine in children with BJHS was 4.5 times higher than healthy children ( *P* = 0.001).

**Conclusion:**

This study showed a significant correlation between BJHS and headache (especially migraine) in children and adolescents.

## Background

Benign Joint Hypermobility Syndrome (BJHS) is a benign hereditary connective tissue disorder in which musculoskeletal symptoms present in the absence of other rheumatic disorders such as Ehlers-Danlos syndrome(EDS), Marfan syndrome, SAPHO( synovitis, acne, pustulosis, hyperostosis and osteitis) and osteogenesis imperfecta [1–5].

BJHS is more common in girls and its prevalence decreases with age. African and Asian children are more hypermobile than Caucasians. A positive family history of hypermobility is common. BJHS is a condition that affects the joints, causing them to move beyond their normal range of motion [2, 3].

BJHS is commonly seen in children and adolescents, and it is estimated to affect 10–25% of the population. BJHS is typically characterized by generalized joint hypermobility, joint pain, and musculoskeletal pain [1–5].

However, recent studies have highlighted the potential association between BJHS and various comorbidities, including gastroesophageal reflux, irritable bowel syndrome and focal hyperhydrosis [2–4].

Different types of headache disorders including migraine, intra- cranial hypotension, Arnold Chiari malformation-type 1, coat-hanger headaches, carotid dissections, cervicogenic headaches, temporomandibular joint disorders (TMDs), and medication overuse headaches have been associated with EDS [4, 7]. There are very few studies on the prevalence of headache in children with BJHS.

Prevalence of headache increases throughout childhood with a peak at 11–13 years old in both sexes. Both migraine and tension type headache (TTH) are the most predominant headaches. It was reported that 6.1 to 13.6% of children have migraine and 9.8 to 24.7% have TTH. Migraine is more disabling and required more medication use than TTH. Ratio of girls to boys in all headaches is 1.5:1 while it is 1.7:1 in migraines. The male to female ratio is 1:3 for migraine and 4:5 for TTH after 12 years of age [5–8].

Headache may be caused by many intracranial conditions such as infectious and inflammatory diseases, brain lesions, vascular abnormalities, high blood pressure, and electrolyte imbalance. Also, multiple extracranial causes such as myositis or myalgia, temporomandibular joint arthritis or TMDs( following rheumatic diseases or secondary to bruxism) or radicular pain may cause headache. Mental disorders may also cause headaches [9–11]. All these cases have become more complicated in recent years during COVID-19 pandemic [12]. This study aimed to investigate the prevalence and subtypes of headache in children and adolescents with BJHS.

## Materials and methods

This school-based observational study was conducted using a case-control method during 2021 to 2023 on children and adolescents aged 7 to 16 years old in Isfahan, Iran. Random sampling was done in two stages by stratified cluster and simple random methods. In the first stage, 4832 students were selected and examined by a pediatric rheumatologist for BJHS using Beighton criteria for Joint hypermobility. In the second stage, all cases with BJHS were included in the case group and among the students without BJHS, 912 students were selected by simple random method and classified into the control group (age- and sex- matched).

Data were collected using Child Headache-Attributed Restriction, Disability, and Social Handicap and Impaired Participation (HARDSHIP) questionnaires for child and adolescent [13]. These questionnaires were used to evaluate the prevalence of headache and diagnostic tool to differentiate among types of headache and their impact on quality of life by using questions about headache frequency and duration, headache characteristics, associated symptoms and medication.

Then both groups were checked for headache using the International Classification of Headache Disorders (ICHD-III), and types of headache were categorized into migraine, tension type headache, and unclassified [14].

Quantitative variables were reported by the mean and the standard deviation (SD), and qualitative variables by number (%). The prevalence of headache was then compared between two groups by Fisher^,^s exact test. The difference between the mean of qualitative variables was evaluated by an independent sample t-test. P values less than 0.05 were considered statistically significant. Statistical analysis was carried out in SPSS version 27.

## Results

In this case control study, 798 patients with BJHS and 912 healthy children were evaluated. The average age was 10.3 ± 3.1 years (range 7–16) and 45.7% were Male (F = 928, M = 782). 76% subjects aged 7–11 years and 23.3% subjects aged 12–16 years were recruited, of which 414 subjects experienced headache (Table 1).

**Table 1:** Prevalence of headache stratified by age and gender in case (with BJHS) and control groups

Age group	Headache	Case, *n* = 798		Control, *n* = 912		*n* (%)	Total *N* = 1710
		Male	Female	Male	Female		
7–11 y	YES	88(14.5)	108(17.9)	41(5.8)	47(6.6)	284(16.6)	1312(76.7)
	NO	192(31.7)	217(35.9)	279(39.5)	340(48.1)	1028(601)	
12–16 y	YES	41(21.2)	55(28.5)	17(8.3)	17(8.3)	130(7.6)	398(23.3)
	NO	44(22.8)	53(27.5)	80(39.0)	91(44.4)	268(15.7)	
Total		365(21.3)	433(25.3)	417(24.4)	495(28.9)		1710(100.0)

As shown in Table 2, there was a statistically significant difference between headache and age in both case and control groups. The occurrence of headache in the age group of 7–11 years in the case group(with BJHS) was 3.37 times more than the control group (*P*-value = 0.001); also, in the age group of 12 to 16 years, the occurrence of headache in the case group was about 5 times more than the control group (*P*-value = 0.001).

**Table 2:** Distribution of headache group in case(with BJHS) and control group by age

Headache	Age groups										
	7–11, *n* = 1312					12–16, *n* = 398					
	Case	Control	*P*-value	OR	95%CI	Case	Control	*P*-value	OR	95%CI	
YES	196	88	0.001	3.37	2.52–4.51	96	34	0.001	4.97	3.06–8.16	
NO	409	619				97	171				
Total	605	707				193	205				

As depicted in Table 3, the probability of headache in children aged 7–11 with BJHS was 3.7 times lower than in children aged 12–16 with BJHS(*P*-value = 0.001). On the other hand, there was no statistically significant difference in the probability of headache in healthy children aged 7–11 years compared to healthy children aged 12–16 years (*P*-value = 0.125).

**Table 3:** Relationship of headache in case(with BJHS) and control group by age

Headache	Case					Control				
	Age group 7–11 12–16		*P*- value	OR	95%CI	Age group 7–11 1216–		*P*- value	OR	95%CI
YES	196	96	0.001	0.27	0.186-0.389	88	34	0.125	0.71	0.457–1.136
NO	409	97				619	171			

In this case-control study of 798 patients with BJHS diagnosed using the Beighton criteria and found that 292 (37%) suffered from headache compared to122 (13.38%) in a healthy control group (Table 4). Migraine was the most common headache type reported of total cases. The probability of headache in children with BJHS was 3.7 times higher than in healthy children (OR = 3.7, *P*-value = 0.001, 95%CI: 2.924–4.785).Furthermore, the probability of migraine in children with BJHS was 4.5 times higher than healthy children (OR = 4.5, *P*-value = 0.001, 95%CI: 3.35–6.27). Furthermore, comparing the occurrence of all headaches according to gender revealed that the probability of occurrence of headache in boys was 1.5 times that of girls, this difference was statistically significant (OR = 1.5, *P*-value = 0.033, 95%CI: 1.013–2.250).

**Table 4:** Types of headache among case(with BJHS) and control group

Type of Headache	Case, *n* = 798			Control, *n* = 912			
	Male, *n* = 365	Female, *n* = 433	Total	Male, *n* = 417	Female, *n* = 495	Total	OR; *P*-value
Migraine	82(22.47)	120(27.71)	202(25.31)	30(7.19)	33(6.67)	63(6.91)	4.5; 0.001
TTH	27(7.40)	17(3.39)	44(5.51)	21(5.04)	25(5.05)	46(5.04)	1.0; 0.664
Other	20(5.48)	26(6.00)	46(5.76)	7(1.68)	6(1.21)	13(1.43)	4.2; 0.001
Total	129(34.34)	163(37.64)	292(36.59)	58(13.91)	64(12.93)	122(13.38)	3.7; 0.001

TTH: tension type headache; OR; Odds ratio

## Discussion

This case-control study showed that the occurrence of headache in children with BJHS was more than the control group and the probability of headache in children with BJHS was 3.7 times higher than in healthy children. Migraine was the most common headache type reported of total cases. Furthermore, the probability of migraine in children with hypermobility was 4.5 times higher than healthy children. Comparing the occurrence of all headaches according to gender revealed that the probability of occurrence of headache in boys was 1.5 times more than of girls. The occurrence of headache in children with hypermobility disorders was more than the control group (P-value = 0.001), and the probability of headache in children aged 7–11 with hypermobility was 3.7 times lower than in children aged 12–16 with hypermobility (P-value = 0.001).

Our results in healthy children (control group) were in line with the Indian study that showed migraine was most prevalent in their cohort followed by TTH. Our results were in line with a Chinese study that showed migraines were more common than any other headache subtypes. The prevalence of migraine in our result (healthy children) was approximately 7%, which is comparable to Kuwaiti children and adolescents studies [15]. Occurrence of headache in our study was less than previous study in this region; the prevalence of primary headaches was reported 9.68%, in the age 9–15 years old in China [16], 24.4% in the age 7–15 in Jordan [17], 25.5% in school children aged 7–14 years old in India [18], 29.1% among school children in Korea [19] and 46.2% in children aged 6–10 in Turkey [20].

The prevalence and burden of primary headache disorders in children and adolescents is a significant public health concern. Primary headache disorders are the most common type of headache disorders and do not have an underlying cause but are independent conditions themselves. Studies have shown that primary headache disorders, such as migraine and tension-type headaches, are highly prevalent in the pediatric population [21, 22]. The prevalence rates are vary depending on dissimilar cultural, genetic, environmental factors, ethnological, cultural and geographical components or methodological dissimilarities or applied diagnostic criteria. Methodological differences in the number of enrolled headache patients, the patient selection criteria, the different age ranges, and the sources of information used (parents or child) and symptom collections probably accounted for differences in the results [5–8].

Migraine, in particular, is the most common primary headache disorders in children. It is estimated that about 5–10% of school-aged children and up to 20% of adolescents experience migraine attacks [23].

Headache (especially migraine attacks) can greatly impact a child’s quality of life, leading to school absences, decreased academic performance, social isolation, and limitations in activities and participation [22–26]. Migraine can affect every domain of life and is associated with poor quality of life and significant disability, if it is not adequately treated. Migraine is an extremely common disorder that geographical, social and economic differences between the different ethnic groups should be mitigated. The recent COVID-19 pandemic has made this situation more complicated [26]. The burden of primary headache disorders in children and adolescents extends beyond the individual and affects families, healthcare systems, and society as a whole. Headaches can lead to increased healthcare utilization and costs, as well as decreased productivity for parents who may need to take time off work to care for their child [23–26].

Tension-type headaches are also prevalent in children and adolescents, with about 15–20% of children experiencing this type of headache. While tension-type headaches are generally less severe than migraines, they can still cause significant distress and impairment in daily functioning.

Furthermore, the long-term implications of primary headache disorders in children are noteworthy. Research has indicated that children with recurrent headaches are at higher risk of developing chronic headaches in adulthood. Therefore, early intervention and appropriate management of primary headache disorders in children and adolescents are important to preventing long-term disability and improving their overall well-being [24].

Strengths of our research is that based on our knowledge it is the first of its kind and it is a novel conception. Also, we used a larger sample. Limitation was the recall bias, which is usual in most of the studies by questionnaire.

## Conclusion

The association between BJHS and headaches in children necessitates increased awareness among healthcare professionals. Further research is needed to elucidate the underlying mechanisms, establish diagnostic criteria, and develop personalized management strategies for this specific population.

## Data Availability

The data are available on request to the corresponding author.

## Abbreviations

BJHS: Benign joint hypermobility syndrome
HARDSHIP: child and adolescent child headache-attributed restriction Disability, and social handicap and impaired participation questionnaire
ICHD: International classification of headache disorders
TTH: Tension type headache
